# Supplementary material for: Expanding two-way texting for post-operative follow-up: A cost analysis of the implementation and scale-up in routine voluntary medical male circumcision settings in South Africa
Source: PLOS Glob Public Health. 2025 Apr 22;5(4):e0004049. doi: 10.1371/journal.pgph.0004049 (PMC12013885; doi:10.1371/journal.pgph.0004049)
Supplement: S2 Table — (DOCX) [file pgph.0004049.s002.docx]

**CHEERS 2022 Checklist**

| **Topic** | **No.** | **Item** | **Location where item is reported** |
| --- | --- | --- | --- |
| **Title** | | | |
| Title | 1 | Identify the study as an economic evaluation and specify the interventions being compared. | Page 1 |
| **Abstract** |  |  |  |
| Abstract | 2 | Provide a structured summary that highlights context, key methods, results, and alternative analyses. | Page 2 |
| **Introduction** |  |  |  |
| Background and objectives | 3 | Give the context for the study, the study question, and its practical relevance for decision making in policy or practice. | Page 2-3 |
| **Methods** | | | |
| Health economic analysis plan | 4 | Indicate whether a health economic analysis plan was developed and where available. | Not applicable |
| Study population | 5 | Describe characteristics of the study population (such as age range, demographics, socioeconomic, or clinical characteristics). | Page 2-5 |
| Setting and location | 6 | Provide relevant contextual information that may influence findings. | Page 2-5 |
| Comparators | 7 | Describe the interventions or strategies being compared and why chosen. | Page 2; Page 5-7 |
| Perspective | 8 | State the perspective(s) adopted by the study and why chosen. | Page 6 |
| Time horizon | 9 | State the time horizon for the study and why appropriate. | Page 4-5 |
| Discount rate | 10 | Report the discount rate(s) and reason chosen. | Page 6 |
| Selection of outcomes | 11 | Describe what outcomes were used as the measure(s) of benefit(s) and harm(s). | N/A |
| Measurement of outcomes | 12 | Describe how outcomes used to capture benefit(s) and harm(s) were measured. | N/A |
| Valuation of outcomes | 13 | Describe the population and methods used to measure and value outcomes. | N/A |
| Measurement and valuation of resources and costs | 14 | Describe how costs were valued. | Page 6-7 |
| Currency, price date, and conversion | 15 | Report the dates of the estimated resource quantities and unit costs, plus the currency and year of conversion. | Page 6-7 |
| Rationale and description of model | 16 | If modelling is used, describe in detail and why used. Report if the model is publicly available and where it can be accessed. | N/A |
| Analytics and assumptions | 17 | Describe any methods for analysing or statistically transforming data, any extrapolation methods, and approaches for validating any model used. | N/A |
| Characterising heterogeneity | 18 | Describe any methods used for estimating how the results of the study vary for subgroups. | N/A |
| Characterising distributional effects | 19 | Describe how impacts are distributed across different individuals or adjustments made to reflect priority populations. | N/A |
| Characterising uncertainty | 20 | Describe methods to characterise any sources of uncertainty in the analysis. | Page 7 |
| Approach to engagement with patients and others affected by the study | 21 | Describe any approaches to engage patients or service recipients, the general public, communities, or stakeholders (such as clinicians or payers) in the design of the study. | Page 4-5 |
| **Results** | | | |
| Study parameters | 22 | Report all analytic inputs (such as values, ranges, references) including uncertainty or distributional assumptions. | Page 8-11; Supplementary material, S1 Table |
| Summary of main results | 23 | Report the mean values for the main categories of costs and outcomes of interest and summarise them in the most appropriate overall measure. | Page 8-10 |
| Effect of uncertainty | 24 | Describe how uncertainty about analytic judgments, inputs, or projections affect findings. Report the effect of choice of discount rate and time horizon, if applicable. | Page 10; Figure 2 |
| Effect of engagement with patients and others affected by the study | 25 | Report on any difference patient/service recipient, general public, community, or stakeholder involvement made to the approach or findings of the study | None |
| **Discussion** | | | |
| Study findings, limitations, generalizability, and current knowledge | 26 | Report key findings, limitations, ethical or equity considerations not captured, and how these could affect patients, policy, or practice. | Page 12-14 |
| **Other relevant information** | | | |
| Source of funding | 27 | Describe how the study was funded and any role of the funder in the identification, design, conduct, and reporting of the analysis | Page 14 |
| Conflicts of interest | 28 | Report authors conflicts of interest according to journal or International Committee of Medical Journal Editors requirements. | Page 14 |

***From:*** Husereau D, Drummond M, Augustovski F, Bekker-Grob E de, Briggs AH, Carswell C, et al. Consolidated Health Economic Evaluation Reporting Standards (CHEERS) 2022 Explanation and Elaboration: A Report of the ISPOR CHEERS II Good Practices Task Force. Value in Health. 2022 Jan 1;25(1):10–31.
