# Supplementary material for: Expanding two-way texting for post-operative follow-up: A cost analysis of the implementation and scale-up in routine voluntary medical male circumcision settings in South Africa
Source: PLOS Glob Public Health. 2025 Apr 22;5(4):e0004049. doi: 10.1371/journal.pgph.0004049 (PMC12013885; doi:10.1371/journal.pgph.0004049)
Supplement: Attachment — Submitted filename: Response To Reviewers.docx [file pgph.0004049.s004.docx]

March 15, 2025

Re: PGPH-D-24-02748

To the PLOS Global Public Health Editorial Office:

Thank you very much for the helpful review and for your understanding in providing us with the extension. We sincerely appreciate the thoughtful comments and feedback provided by the reviewers which have greatly contributed to improving the quality and clarity of the manuscript to reach our intended readers.

Attached please find the revised version of the manuscript originally titled “What does it cost to expand two-way texting for post-operative follow-up? A cost analysis in routine voluntary medical male circumcision settings in South Africa” (Manuscript ID: PGPH-D-24-02748), which we had previously submitted for review in PLOS Global Public Health.

We have carefully addressed all the reviewers' comments and made the necessary revisions to the manuscript. The table below addresses each point raised by the reviewers, along with the line number from the clean version of the manuscript, when applicable. We believe the revisions have significantly strengthened the manuscript, and hope that the updated version will meets the expectations of the journal.

Thank you again for your time and consideration, please send any additional correspondence concerning this manuscript to: [cfeld@uw.edu](mailto:cfeld@uw.edu)

Sincerely,

Caryl Feldacker on behalf of all authors

| **Reviewer** | **Comment** | **Response / Action Taken** | **Location in Manuscript** |
| --- | --- | --- | --- |
| Reviewer 1 | In the title, it is recommended that the study should reflect which health interventions were being compared. In this study it was the intervention (2wt) which boldly appeared - but not the comparator (routine care). This needs to be reviewed. | The title has been revised accordingly. | Title (lines 1-4) |
|  | It is a standard and requirement to explain whether the study is full economic evaluation or partial evaluation by developing Health Economics Analysis Plans (HEAP) for guiding the conduct of the economic evaluation as per the Consolidated Health Economic Evaluation Reporting Standards (CHEERS) checklist. Can the authors justify why this was not done? | The cost analysis did follow the guidance for health economic evaluations as described by the CHEERS 2022 statement. In this revision, we made clear that this framework and checklist was followed. Clarity was added to the Methods along with a completed CHEERS checklist. | Methods – Costing Methodology (lines 189-191); S2 Table |
|  | Can the authors provide what type of sensitivity analysis was used? One-way? Two-way? This need to be specified and the rational for the choice. | One-way univariate sensitivity analyses were used; we further specified this in the Methods section. | Methods – Objective 3 (lines 257-261) |
|  | Describing the cost in ZAR is important as that of USD, and this has to be provided not only the exchange rate. | Costs were primarily collected in ZAR and converted to USD for analysis. All costs are reported in both ZAR and USD in supplemental materials for those who would like both currencies in the tables. | S1 Table |
|  | The role of the funder in the identification, design, conduct, and reporting of the analysis was not clearly reported. | We revised the Ethics statement to clearly indicate that the funder had no role in the study design, data collection and analysis, decision to publish, or preparation of the manuscript. | Methods – Ethics (lines 267-268) |
|  | There also needs for some editorial issues. The authors should note that "data" are plural. | Thank you, we have carefully reviewed the manuscript to correct grammatical errors. | Throughout |
| Reviewer 2 | Additional evidence that targets scaleup, if any, that would support your conclusion that “2wT should be expanded for eligible males across VMMC and other postoperative contexts in South Africa” would be essential. | We have added an additional discussion paragraph to further explain how this cost analysis provides evidence and increases transparency for further 2wT scale-up | Discussion (lines 402-423) |
|  | Does this conclusion serve clients/adults living in urban, rural, or both? | This analysis found that scaling-up the implementation of 2wT in routine settings, regardless of rurality, will be cost neutral. Though we did not have sufficient data to evaluate the differences in cost by rurality during routine implementation, a prior cost analysis of implementation during the RCT in South Africa (Su et al. 2023) found cost savings were greater in rural areas, indicating cost savings may be higher to clients/adults living in rural areas. However further evaluation is needed to assess whether the difference in savings continues in routine implementation and scale-up. |  |
|  | Do you add a support of evidence that shows the current digital infrastructure of South Africa makes it easy for your conclusion to implement or scale up? At least you should provide evidence on the coverage of clients who can use a basic mobile phone with SMS capability | South Africa has an extremely high level of mobile penetration, with over 99% 3G coverage reported nationally in 2021. We have added this to the discussion. | Discussion (lines 413-415) |
